# Nitrate of Silver in Leucorrhea

**Published:** 1851-12

**Authors:** 


### SURGERY.

*Nitrate of Silver in Leucorrhea.*—Dr. N. B. Anderson, of Louisville, Ky., strongly recommends the application of the solid nitrate of silver to the cervix uteri, and adjacent parts—by means of the speculum—as the true remedy for leucorrhea. Dr. Anderson urges, with much force, the necessity of resorting to the speculum, as the only means of correct diagnosis or treatment, in leucorrhea. He has found that the disease is, almost invariably, connected with an ulcerated condition of the neck of the uterus, and he believes that astringent injections are very *inefficacious* in reaching the seat of the disease, which requires the application of solid caustic. He gives the following account of his experience and practice.

"In the course of the last year I have had an opportunity of treating forty-one cases of leucorrhea, and the results have been so satisfactory that I feel justified in submitting my experience to the profession. I am sure that no physician, after trying the improved method, will ever be satisfied to return to the old practice of tonics and astringent injections. Not that tonics are not useful in their proper place. I have found them highly beneficial in many cases, but they are to be looked upon as adjuvants to the main remedy—the nitrate of silver locally applied. So long as the morbid condition giving rise to the unhealthy secretion continues, constitutional remedies are of no avail. The cause must first be removed, and at a knowledge of this, as has already been remarked, we must arrive by means of the speculum.

"In nine cases out of ten which have come under my treatment, I have found no difficulty in obtaining the consent of the patient to the use of the speculum: the woman is then placed in a horizontal position on a lounge or bed, the hips elevated higher than the head; a loose sheet is thrown over her person, in which is an aperture sufficient to admit the speculum; (that which I use is glass;) it is well lubricated, and then insinuated as far as possible into the vagina, following the axis of the pelvis;—the os uteri readily descends into the mouth of the instrument, inclining backwards and downwards, which, by depressing the handle, will remain in the centre of the instrument. In this position, before a window, or with the use of a candle, the organ can viewed to its full extent, and the ulcerated surface found. In nearly every case it presents the same appearance. The neck is found flabby, swollen, and of a pale red and bluish cast—the anterior and posterior lips being much elongated, with the greater portion of the neck ulcerated to some extent. In about one-half of the cases, the ulceration is within, as well as without the neck, and extends some distance into the cavity of the uterus; whilst in the remaining half, it is limited to an area of one-third of the neck, and anterior and posterior lips, the cavity not partaking of the ulceration. In every case, the ulcerated surface is to some extent the same, with but little sensibility about the parts; no pain is ever felt on applying the caustic freely, both around the neck and within the uterine cavity, for the mouth is always open sufficiently to admit of its introduction.

"In the first place, the parts are cleansed with a soft sponge attached to a whale-bone handle, in mop shape, after which the nitrate of silver, in the solid form, is applied very freely to every part diseased. This operation is repeated according to indications, from three to seven days apart, and during the interval, cold water injections several times a day are to be used conjointly with tonics. In some cases I have used the speculum as often as ten times, but in the great majority of cases, it is not necessary to resort to it often.

"Those cases in which the ulceration was seated within the neck and cavity of the uterus occurred in weakly females, who had borne a great number of children, and some with the aid of forceps. In unmarried ladies, it was always seated on the external parts of the neck, and the anterior lip. In the cases in which the cavity was implicated, the free use of the silver was not found to have interfered in the least with the catamenia.

"In almost every case where a female does not bear children, I have found fluor albus to be present;—unquestionably this disease is a frequent cause of sterility; the ulceration of the neck and cavity interfering with the intromission of the semen, which passes away with the profuse discharge."—*Abridged from Western Jour. Med. and Surgery.*

